# Effects of different gastrointestinal reconstruction techniques on nutrition, anemia, and quality of life in laparoscopic distal gastrectomy for gastric cancer

**DOI:** 10.1590/acb370408

**Published:** 2022-07-15

**Authors:** Bu Jun, Li Nian, He Shan, Yuan Hong-Jun, Deng Heng-Yi, Wen Wu, Yang Xiao-Yan

**Affiliations:** 1 PhD. Chengdu Second People’s Hospital – Department of General Surgery – Chengdu, China.; 2 MD. Chengdu Second People’s Hospital – Department of General Surgery – Chengdu, China.; 3 MD. Chengdu Second People’s Hospital – Department of Digestive Medicine – Chengdu, China.; 4 PhD. Chengdu Second People’s Hospital – Department of Digestive Medicine – Chengdu, China.

**Keywords:** Stomach Neoplasms, Laparoscopy, Gastrectomy, Quality of Life

## Abstract

**Purpose:**

To explore the effect of different gastrointestinal reconstruction techniques on laparoscopic distal gastrectomy of gastric cancer on the nutritional and anemia status, and quality of life (QoL) of patients.

**Methods:**

Eligible patients were randomly divided into three groups (n=36/group): Billroth I anastomosis group, Billroth II combined with Braun anastomosis group, and Roux-en-Y anastomosis group. Related indicators were compared and analyzed.

**Results:**

The general data were comparable among the three groups (all *P>*0.05). Among the surgical-related indicators and postoperative recovery indicators, only the comparison of the operation time was statistically significant (*P*=0.004). The follow-up time was 5~36 months (average 27.9 months). In terms of nutritional and anemia indicators, only the differences in the levels of prealbumin, hemoglobin and serum ferritin in 24 months after operation showed significant differences (*P*=0.015, *P*=0.003, *P*=0.005, respectively). There were no significant differences in hospital readmission rate, overall survival, and QoL among the three groups (all *P>*0.05).

**Conclusions:**

In laparoscopic gastrectomy for stage II~III distal gastric cancer, Billroth I anastomosis has shorter operation time than Billroth II combined with Braun anastomosis and Roux-en-Y anastomosis and advantages in the improvement of nutritional status and anemia recovery.

## Introduction

Gastric cancer is a common malignant tumor of the digestive tract. Especially in China, the incidence of gastric cancer in malignant tumors ranked second, and the mortality ranked second^1^,^2^. Up to now, surgical treatment is still the most important means to achieve radical cure of gastric cancer^3^. However, the common nutritional disorders and anemia after gastrectomy are often ignored by people^4^. When the tumor condition of patients with distal gastric cancer allows the use of different reconstruction methods to complete the operation, which method is more conducive to the improvement of postoperative nutritional status and anemia status, more conducive to improve the quality of life (QoL) of patients, there is still a lack of conclusion^5^-^8^.

In this study, randomized controlled trial (RCT) was used to compare the postoperative outcomes of three different digestive tract reconstruction methods in patients with distal gastric cancer after laparoscopic surgery, and to study its impact on nutritional status, anemia status and Qol of these patients.

## Methods

### Participants

From June 2017 to June 2019, a total of 108 patients (estimated loss of follow-up rate of 5%) with gastric cancer who were admitted to the general surgery department were enrolled. The patients were assigned to three groups (n=36 each) using the random number table method: Billroth I anastomosis group, Billroth II combined with Braun anastomosis group, and Roux-en-Y anastomosis group. All patients were followed up until June 2021.This study was reviewed and approved by the Institutional Review Board (IRB) (Project No.: 2017032).

### Inclusion and exclusion criteria

The inclusion criteria were as follows: a diagnosis of gastric cancer by a preoperative endoscopy pathological biopsy, preoperative TNM–TNM classification system of the National Comprehensive Cancer Network (NCCN) 2017 Clinical Practice Guidelines for gastric cancer–staging of II~III, gastroscopy and computed tomography (CT) confirmed that the tumor was located in the lower 1/3 area of the stomach, age ≥18 and ≤81 years old, no emergency surgery, no preoperative radiotherapy or chemotherapy, American Society of Anesthesiology score grades I~III, and preoperative informed consent obtained.

The exclusion criteria were as follows: severe cardiopulmonary diseases or other severe organ dysfunction, uncontrolled hyperthyroidism or hypothyroidism, merging other malignant tumors, autoimmune diseases, long-term glucocorticoid treatment, pregnant or lactating women, and no hope for survival, dying or irreversible coma patients.

### Methods

#### Principles of surgery and preoperative preparation

All surgeries were performed by the same team of surgeons, following the principle of radical surgery of tumors. TNM staging of gastric cancer was determined by abdominal enhanced CT within two weeks before operation. The perioperative management followed the concept of enhanced recovery surgery after gastrectomy for gastric cancer^9^, and standardized use of antibiotics.

#### Surgical mode

The operation process was in accordance with the operation guidelines for laparoscopic gastric cancer surgery (2016 Edition)^10^ and the expert consensus on quality control of laparoscopic radical gastrectomy in China (2017 Edition)^11^, and D2 lymph node dissection was performed. All patients were performed laparoscopic assisted distal gastrectomy through right anterior approach, and mechanical anastomosis was performed with the same brand of anastomotic instruments under small incision of abdominal wall. During the surgery, if the tumor could not be removed by laparoscopy, it should be converted to laparotomy in time. Three groups of patients underwent classic Billroth I anastomosis, Billroth II combined with Braun anastomosis and Roux-en-Y anastomosis.

#### Nutritional therapy^12^,^13^

The patients with moderate to severe malnutrition were given nutritional support for one week before operation. Postoperative parenteral nutrition (PN) support was provided according to the standard of relatively low nitrogen (0.17 g / (kg d)) and low calorie (nonprotein calorie 83.7kj / (kg d)). All patients were infused via central vein (subclavian vein or internal jugular vein) by catheter. The infusion time was 12~16 hours per day, and the speed was controlled by infusion pump.

#### Observed indicator

General data, surgical and therapeutic indicators are in Table 1. Postoperative recovery indicators are in Table 2.

**Table 1:** General clinical data.

		Billroth I group	Billroth II+Braun group	Roux-en-Y group
Gender(M/F)		21/15	18/18	22/14
Age(y)		66.22 ± 9.03	65.64 ± 8.59	63.86 ± 9.22
BMI(kg/m^2^)		22.09 ± 2.64	22.47 ± 2.61	21.00 ± 3.39
cTNM stage(n)	II	25	29	27
	III	11	7	9
ASA classification (n)	I	11	9	8
	II	19	20	23
	III	6	7	5
Time of operation (min)		153.06 ± 23.28	170.28 ± 22.36	165.83 ± 21.70*
Intraoperative blood loss (mL)		59.44 ± 27.04	57.22 ± 25.81	61.11 ± 21.75
Conversion to open (yes/no)		3/33	4/32	2/34
Number of lymph node (n)		28.64 ± 6.10	26.22 ± 5.26	25.78 ± 5.90
pTNM stage(n)	II	15	14	11
	III	17	19	23
	IV	4	3	2
Degree of pathological differentiation(n)	high	5	3	4
	moderate	14	8	11
	poor	17	25	21
Perioperative blood transfusion (n)		7	5	9
Postoperative chemotherapy alone (yes/no)		19	20	15
Postoperative chemoradiotherapy (n)		5	4	7

BMI: body mass index; ASA: American Society of Anesthesiology; Comparison among the three groups: *F=5.708, *P=0.004

**Table 2:** Postoperative recovery indicators.

Postoperative	Billroth I group	Billroth II+ Braun group	Roux-en-Y group	Comparison	
				F (χ^2^) value	*P* value
Time of first flatus (day)	2.69 ± 0.82	2.39 ± 0.84	2.78 ± 0.72	F=1.509	*P*=0.097
SIRS (n)	2	1	2	χ^2^=0.456	*P*=0.796
Incision infection (n)	1	2	1	χ^2^=0.490	*P*=0.783
Pulmonary infection (n)	0	1	1	χ^2^=1.641	*P*=0.440
Urinary tract infection (n)	1	0	1	χ^2^=1.641	*P*=0.440
Reflux esophagitis(n)	6	2	2	χ^2^=3.298	*P*=0.192
Delayed gastric emptying (n)	3	2	2	χ^2^=0.295	*P*=0.863
Bleeding (n)	1	0	0	χ^2^=2.216	*P*=0.330
Vomiting (n)	1	1	2	χ^2^=0.490	*P*=0.783
Obstruction (n)	1	0	2	χ^2^=2.830	*P*=0.243
Hospital stay time (day)	11.39 ± 1.86	11.53 ± 1.78	11.06 ± 1.60	F=0.693	*P*=0.503

SIRS: systemic inflammatory response syndrome.

#### Nutritional indicators and anaemic indicators

Albumin (ALb), prealbumin (PA), body weight, red blood cell (RBC), hemoglobin (Hb), serum ferritin (Fer), serum vitamin B_12_ (VitB_12_), and serum folate (Fol) were measured one week before operation and one month, six months, 12, and 24 months after operation.

#### Clinical outcome indicators

Incidence of hospital readmission within 30 days, overall survival (OS), and disease-free survival (DFS).

### Data processing and statistical methods

The database was built using Excel 2007 software, and statistical analyses were performed using Statistical Package for the Social Sciences (SPSS) 19.0 software (International Business Machines Corporation). The survival curve was plotted by GraphPad Prism 7.0 software (Graphpad^®^), and followed by the intention-to-treat (ITT) principle. The measurement data were presented as means ± standard deviation ( *X* ± s), and the differences in normal distribution and homogeneity of variance were compared by t-test or analysis of variance. The count data were compared by *X* ^2^ test. Survival analysis was performed using a Kaplan-Meier curve, and log-rank test was used to compare the variables. All statistical tests were performed by two-tailed test. The power of the test was calculated by 1-β=0.9 (β=0.1), and *P*<0.05 was considered statistically significant.

## Results

### General data and surgery-related indicators

During this study, a total of 134 patients with gastric cancer were evaluated for eligibility. Of them, 26 patients, as not meeting the inclusion criteria, were excluded. Finally, 108 were enrolled and assigned to three groups (n=36) according to random number table. There were 61 male patients and 47 females, with an age range of 34~76 years old, and average of 65.2 years old. Analysis found that the general information and surgical indicators of three groups of patients, only the difference of operation time was statistically significant (*P* = 0.004), the other indicators were not statistically significant (all *P*>0.05, see Table 1 for details). The patients were followed up for 5~36 months with average of 27.9 months.

### Recovery indicators

Analysis found that the recovery indicators of three groups of patients were not statistically significant (all *P*>0.05, see Table 2 for details), and there was no postoperative intestinal leakage in the three groups of patients.

### Nutritional indicators and anaemic indicators

Analysis found the nutritional indicators and anaemic indicators of three groups of patients. Only the differences in the levels of PA, Hb and Fer in 24 months after operation showed significant (*P*= 0.015, *P*= 0.003, *P*= 0.005, respectively). The other indicators were not statistically significant (all *P*>0.05, Table 3).

**Table 3:** Nutritional indicators and anaemic indicators.

	Time	Billroth Igroup	Billroth II+Braun group	Roux-en-Ygroup
Alb (g/L)	preoperative	36.89 ± 2.83	36.42 ± 2.48	36.64 ± 2.53
	1 month	35.97 ± 2.01	36.39 ± 2.26	36.14 ± 1.91
	6 months	36.50 ± 1.87	36.58 ± 2.20	36.33 ± 2.11
	12 months	37.64 ± 1.97	37.19 ± 2.20	37.11 ± 2.14
	24 months	37.64 ± 2.11	37.47 ± 2.09	37.31 ± 2.00
PA (mg/L)	preoperative	256.19 ± 22.50	256.47 ± 26.11	259.14 ± 28.80
	1 month	231.53 ± 21.01	227.89 ± 23.89	229.28 ± 27.49
	6 months	244.94 ± 19.36	238.61 ± 25.31	235.58 ± 27.00
	12 months	249.17 ± 21.53	244.80 ± 27.48	243.11 ± 24.32
	24 months	270.06 ± 27.81	258.61 ± 30.05	250.42 ± 27.02•
Weight (kg)	preoperative	59.64 ± 7.78	60.78 ± 7.66	57.58 ± 9.65
	1 month	57.75 ± 6.94	58.92 ± 7.66	56.42 ± 9.48
	6 months	57.97 ± 6.79	59.25 ± 7.37	56.78 ± 9.41
	12 months	60.28 ± 6.54	59.75 ± 7.30	57.39 ± 9.14
	24 months	60.44 ± 6.44	60.14 ± 7.06	57.83 ± 8.88
RBC (*10^9^/L)	preoperative	4.34 ± 0.50	4.38 ± 0.57	4.26 ± 0.48
	1 month	4.39 ± 0.49	4.51 ± 0.48	4.46 ± 0.54
	6 months	4.44 ± 0.46	4.49 ± 0.50	4.36 ± 0.47
	12 months	4.30 ± 0.45	4.19 ± 0.43	4.08 ± 0.51
	24 months	4.22 ± 0.45	4.13 ± 0.37	4.00 ± 0.48
Hb (g/L)	preoperative	120.06 ± 15.89	113.47 ± 16.29	115.97 ± 17.91
	1 month	118.67 ± 15.01	112.61 ± 14.09	116.00 ± 16.39
	6 months	120.72 ± 13.98	115.33 ± 15.30	118.86 ± 15.73
	12 months	122.58 ± 13.97	116.50 ± 13.33	118.56 ± 15.67
	24 months	124.00 ± 11.89	113.50 ± 13.16	116.14 ± 14.49▲
Fer (ng/mL)	preoperative	102.01 ± 21.64	99.95 ± 15.37	96.76 ± 23.20
	1 month	93.95 ± 18.54	90.23 ± 15.90	92.32 ± 16.97
	6 months	87.01 ± 15.74	88.51 ± 15.37	90.93 ± 16.74
	12 months	83.96 ± 14.99	82.18 ± 14.79	81.13 ± 14.42
	24 months	79.78 ± 13.17	70.23 ± 13.93	73.35 ± 9.95■
VitB_12_ (pg/mL)	preoperative	553.33 ± 160.18	576.64 ± 134.98	540.97 ± 117.96
	1 month	558.89 ± 152.62	576.64 ± 127.54	540.97 ± 125.17
	6 months	572.78 ± 145.62	593.31 ± 121.48	557.64 ± 109.57
	12 months	564.44 ± 147.66	582.19 ± 122.42	543.75 ± 122.56
	24 months	536.67 ± 132.74	532.19 ± 140.98	507.64 ± 139.03
Fol (ng/ml)	preoperative	7.50 ± 3.11	7.94 ± 3.69	8.34 ± 4.14
	1 month	8.17 ± 2.92	8.59 ± 4.01	9.06 ± 4.29
	6 months	8.82 ± 3.60	9.06 ± 4.05	9.42 ± 4.16
	12 months	8.26 ± 3.51	8.37 ± 3.35	7.87 ± 3.17
	24 months	7.98 ± 3.68	7.47 ± 3.06	7.03 ± 2.89

ALb: albumin; PA: prealbumin; weight: body weight; RBC: red blood cell; Hb: hemoglobin; Fer: serum ferritin; VitB_12_: serum vitamin B_12_; Fol: serum folate; comparison among the three groups:

• F=4.367; ^•^ *P*=0.015;

▲ F=6.140; ^▲^ *P*=0.003;

■ F=5.487; ^■^ *P*=0.005.

**Figure 1:**
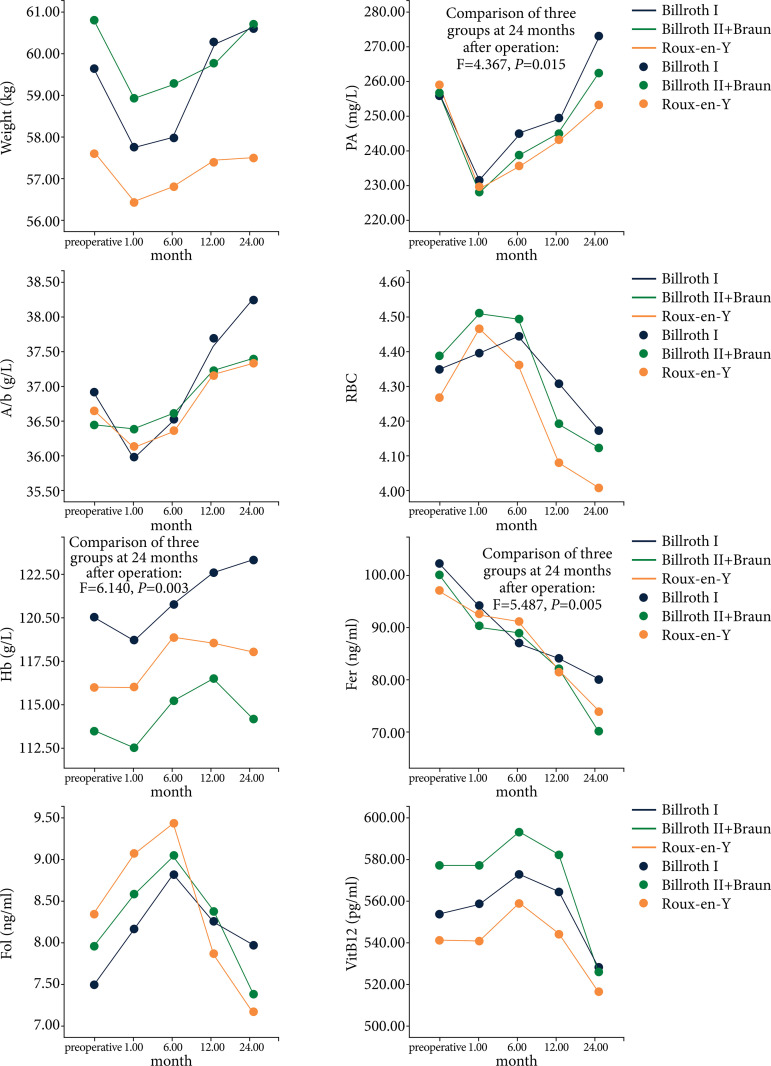
Change curve of nutritional indicators and anaemic indicators.

ALb: albumin; PA: prealbumin; weight: body weight; RBC: red blood cell; Hb: hemoglobin; Fer: serum ferritin; VitB_12_: serum vitamin B_12_; Fol: serum folate.

According to the trend chart of nutritional indicators and anaemic indicators of the three groups of patients during the follow-up period, they can be generally divided into three trends: weight, PA, ALb and Hb showed a downward trend at first, and gradually increased at one month after operation. And RBC, Fol and VitB_12_ showed an increasing trend at first and a decreasing trend at six months after operation. However, Fer showed a downward trend after operation (Fig. 1).

### Clinical outcomes indicators

**Figure 2:**
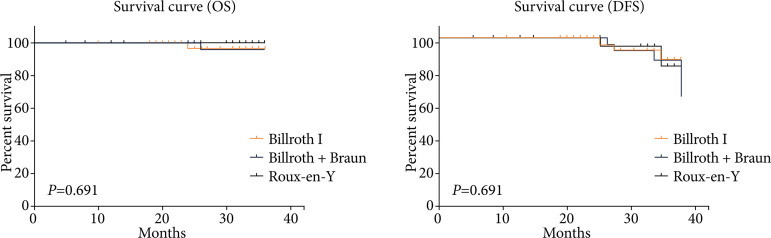
Comparison of survival curves among the three groups.

OS: overall survival; DFS: disease-free survival.

There were no significant differences in unplanned hospital readmission rate, OS, DFS and QoL among the three groups (all *P* > 0.05, Table 4 and Fig. 2).

**Table 4:** Clinical outcomes indicators.

		Billroth I group	Billroth II +Braun group	Roux-en-Y group	Comparison	
					χ^2^ value	*P* value
Hospital readmission (n)		1	0	1	0.490	0.783
QoL (n)	Live independently	30	30	32	4.064	0.851
	Need assistance	2	1	1		
	Tumor-related death	1	1	0		
	Death of other causes	0	1	1		
	Loss to follow-up	3	3	2		

QoL: quality of life.

## Discussion

Gastric cancer mainly occurs in gastric antrum and distal lesser curvature of stomach. After distal gastrectomy, iron absorption and endogenous factor secretion of gastric parietal cells decreased^14^. The described situation will lead to anemia in patients, and then the blood carrying capacity of the patients will be weakened^15^. Subsequently, the organ dysfunction will occur, and the QoL will be affected^15^,^16^. This study suggests that the operation time of Billroth I anastomosis is better than Billroth II combined with Braun and Roux-en-Y anastomosis in laparoscopic gastrectomy for stage II~III distal gastric cancer, and it has advantages in the improvement of long-term nutritional status and recovery of anemia after operation. At the same time, there was no significant difference in OS, DFS and QoL among the three anastomoses.

Among the surgery-related indicators, the specific difference was that the operation time of Billroth I group was shorter than that of Billroth II combined with Braun group and Roux-en-Y group (*P*=0.004). The reason may be that Billroth II combined with Braun and Roux-en-Y have more anastomosis than Billroth I, and the operation procedure is increased, which leads to the extension of operation time. This is consistent with the results of other similar studies^17^-^20^.

In this study, there was no significant difference between the three groups in terms of the postoperative complications. At present, in the study of different reconstruction methods of laparoscopic gastrectomy, few Billroth II combined with Braun anastomosis methods were included in the comparative analysis. In addition, there is no unified result about which one has high incidence of postoperative complications between Billroth I anastomosis and Roux-en-Y anastomosis. In 2019, a RCT study conducted by Ren and Wang^21^ showed that the postoperative complications of Billroth I anastomosis were higher than Roux-en-Y anastomosis in laparoscopic radical gastrectomy for gastric cancer. On the contrary, Nakanishi *et al*.^22^ conducted a control study on 1,014 patients with distal gastric cancer in 2020 and found that the incidence of postoperative complications of Billroth I anastomosis was lower than that of Roux-en-Y anastomosis. Overall, the incidence of reflux esophagitis in patients with Billroth I anastomosis after distal gastrectomy is higher than that in patients with Roux-en-Y anastomosis^5^,^23^.

In perioperative nutritional indicators, the recovery of PA level in the Billroth I group was better than that in the other two groups (*P* < 0.05) at 24 months after operation. In addition, it should be noted that from the change curve of nutritional indicators of three groups of patients, the average values of PA, Alb and Weight decreased at one month after operation, which was once lower than the preoperative level, and showed an upward trend at 6~24 months after operation, and the upward trend was gentle at 12~24 months after operation. Similar studies^24^,^25^ found that the Weight and Alb of gastric cancer patients with different reconstruction methods within three months after operation were lower than those before operation, and recovered to close to the preoperative level after 12 months.

The impact of reconstruction methods on the anemia status of patients is also an important consideration when choosing the anastomosis method^7^. Jeong *et al*.^26^ found that the incidence of anemia in patients with gastric cancer after surgery was as high as 78.3%, and there was no significant difference in anemia of patients with distal gastrectomy within 12 months after different reconstruction methods. In this study, only the recovery of Hb and the decrease of the level of Fer after Billroth I anastomosis were better than those of the other two groups(both *P*<0.05). In addition, this study found that the level of Fer had a downward trend after surgery, while RBC, Fol and VitB_12_ levels were all increased after surgery, and gradually decreased at six months after surgery. Similarly, Lee *et al*.^4^,^24^ found that the incidence of iron deficiency anemia was 31%, and the level of Fer decreased gradually within 24 months after different reconstruction methods of surgery. However, on the contrary, Tang *et al.* 27 conducted a retrospective study on 126 patients with gastric cancer and found that the level of Fer showed a gradual increase trend within the average follow-up time of 15.8 months. In addition, Hu *et al*.^28^ studied 469 cases of distal gastric cancer and found that the VitB_12_ deficiency rate was 15.7% at four years after operation. Therefore, for patients with gastric cancer, postoperative standard, and reasonable supplement of VitB_12_, folic acid and iron is of great clinical significance.

Moreover, the results showed that there was no significant difference in readmission rate, OS, DFS and QoL among the three groups within the follow-up period of 27.9 months (all *P* > 0.05).

This study has several limitations. First, it is not a multi-center study, and the sample size is small. Second, the follow-up time was less than five years. Thus, further research is needed to improve the described limitations to obtain more findings.

## Conclusions

The results of this study suggest that when laparoscopic radical surgery for locally advanced distal gastric cancer is available, Billroth I anastomosis is fast and physiological, which is more conducive to the improvement of long-term nutritional status and the correction and prevention of anemia. In addition, although Roux-en-Y anastomosis and Billroth II combined with Braun anastomosis have certain advantages in short-term postoperative recovery, the long-term QoL of the three anastomosis methods is similar.

### Main points

- There were no significant differences in hospital readmission rate, OS, and QoL among the three different gastrointestinal reconstruction techniques groups of gastric cancer;
- The differences in the levels of PA, Hb and Fer in 24 months after operation showed significant differences among the three groups;
- In laparoscopic gastrectomy for stage II~III distal gastric cancer, Billroth I anastomosis has shorter operation time than Billroth II combined with Braun anastomosis and Roux-en-Y anastomosis.
